# ﻿Description of two new species of the leafhopper genus *Pediopsis* Burmeister (Hemiptera, Cicadellidae, Eurymelinae, Macropsini) from China

**DOI:** 10.3897/zookeys.1149.81434

**Published:** 2023-02-22

**Authors:** Hu Li, Ran-Huai Dai, Michael D. Webb

**Affiliations:** 1 Shaanxi Key Laboratory of Bio-resources, School of Biological Science & Engineering, Shaanxi University of Technology, Qinling-Bashan Mountains Bioresources Comprehensive Development C.I.C., State Key Laboratory of Biological Resources and Ecological Environment of Qinling-Bashan, Shaanxi, 723000, Hanzhong, China Institute of Entomology of Guizhou University Guiyang China; 2 Institute of Entomology of Guizhou University, The Provincial Key Laboratory for Agricultural Pest Management of Mountainous Region, Guizhou, 550025, Guiyang, China Shaanxi University of Technology Hanzhong China; 3 The Natural History Museum, SW7 5BD, London, UK The Natural History Museum London United Kingdom

**Keywords:** Auchenorrhyncha, Homoptera, morphology

## Abstract

Two new leafhopper species of *Pediopsis* Burmeister, *Pediopsisalbopicta* Li & Dai, **sp. nov.** from Hunan and Guizhou provinces of central China and *Pediopsispianmaensis* Li & Dai, **sp. nov.** from Yunnan Province of southwestern China, are described and illustrated. Ambiguity in the original description of *P.bannaensis* Yang & Zhang is discussed, and figures of the female holotype of *P.femorata* Hamilton are provided for the first time. A checklist and key to Chinese species of *Pediopsis* are also given.

## ﻿Introduction

The leafhopper genus *Pediopsis* Burmeister, belonging to the tribe Macropsini of the subfamily Eurymelinae (*sensu*Dietrich and Thomas 2018), was established by Burmeister (1838) as a subgenus of *Bythoscopus* Germar; it was subsequently raised to the level of genus and *Jassustiliae* Germar, 1831 was designated as its type species by Kirkaldy (1903). Many authors (Anufriev 1971; Hamilton 1980; Tishechkin 1997; Cai et al. 2005; Dai and Li 2013; Yang and Zhang 2015; Yang et al. 2016) have described new species or proposed new combinations in the genus worldwide, increasing the number of species to 19, of which six are known from China (Dai et al. 2018). In this paper, two new species of *Pediopsis* from China are described, ambiguity in the original description of *P.bannaensis* Yang & Zhang is discussed, and the status of *P.femorata* Hamilton is commented on. Figures of the female holotype of *P.femorata* for the first time are provided. In addition, a checklist and key to the Chinese species of *Pediopsis* are given.

## ﻿Materials and methods

Specimens studied were collected by netting. External morphology was observed under Olympus SZX7 and BX43 microscopes. Male terminalia preparations were macerated in a boiling solution of 8% NaOH for ~ 5 min. Habitus images of adults were obtained by using a KEYENCE VHX-1000 system. Genitalia drawings were created and edited utilizing Adobe Illustrator CS6 and Photoshop CS6 based on line drawings of specimens.

The higher classification and morphological terminology used in this work follows Hamilton (1980) and Dietrich and Thomas (2018). Body length is measured from the apex of the head to the end of the folded forewings and presented in millimeters (mm).

Type specimens of the new species and other material examined are deposited in the Institute of Entomology, Guizhou University, Guiyang, China (**GUGC**).

## ﻿Systematics

### ﻿Genus Pediopsis

Taxon classificationAnimaliaHemipteraCicadellidae

Burmeister

D0563FF6-5B19-5D05-B350-D5914CA3CC28

Bythoscopus (Pediopsis) Burmeister, 1838: 11.
Pediopsis –Kirkaldy 1903: 214; Hamilton 1980: 902.

#### Type species.

*Jassustiliae* Germar, 1831, by subsequent designation of Kirkaldy (1903).

#### Distribution.

Palaearctic, Oriental, Nearctic, and Australian regions.

#### Remarks.

*Pediopsis* can be distinguished by the following combination of features: head across eyes usually distinctly narrower than pronotum, face wider than long, lora relatively large, pronotum frontally declivous and usually with strongly oblique striations, male pygofer without spines or processes, dorsal connective usually strongly developed. The traditional separation of *Pediopsis* from *Pedionis* (Hamilton 1980) is followed here, but, as more species become known, the two genera may be synonymized. The difficulty in defining *Pediopsis* is apparent from the fact that the genus was keyed out in two places in Hamilton’s (1980) key. At present, the most reliable feature to separate the two genera is the presence or absence of processes or spines on the ventral margin of the male pygofer (absent in *Pediopsis* and present in *Pedionis*).

##### ﻿Checklist to species of *Pediopsis* from China

*P.albopicta* Li & Dai, sp. nov. Figs 1–11. Distribution. China (Hunan and Guizhou provinces).

*P.bannaensis* Yang & Zhang, 2015: 488, figs 29–39. Distribution. China (Yunnan Province), Thailand.

*P.cudraniae* Cai & Wang, 2005: 206, fig. 1. Distribution. China (Shandong Province).

*P.femorata* Hamilton, 1980: 919; Figs 22–26. Distribution. China (Taiwan).

*P.kurentsovi* Anufriev, 1971: 95, figs 4–6; 1976: 133. Distribution. China (Hebei, Heilongjiang provinces), Russia.

*P.ningxiaensis* Dai & Li, 2013: 961, figs 22–31. Distribution. China (Ningxia Province).

*P.tiliae* (Germar, 1831: 14), Hamilton 1980: 903, fig. 62. Distribution. Widespread in Palaearctic region.

*P.pianmaensis* Li & Dai, sp. nov. Figs 12–21. Distribution. China (Yunnan Province).

### ﻿Key to species of *Pediopsis* recorded in China

**Table:** 

1	Fore margin of head and pronotum in dorsal view strongly arched forward (Fig. 22)	***P.femorata***
–	Fore margin of head and pronotum in dorsal view moderately arched forward	**2**
2	Mesonotum with white tip and veins of forewings with white spots (Fig. 1); aedeagal shaft very short (Fig. 10)	***P.albopicta***
–	Mesonotum without white tip and veins of forewings without white spots; aedeagal shaft short to long	**3**
3	Species mainly dark (Fig. 12); forewing with two subapical cells (Fig. 14)	***P.pianmaensis***
–	Species mainly pale, sometimes forewing with distinct brown markings; forewings with two or three subapical cells (Fig. 4)	**4**
4	Dorsal connective with process near midlength or subbasally	**5**
–	Dorsal connective with process absent	**7**
5	Dorsal connective with well-developed process near midlength, caudodorsally twisted Y-shaped (see Yang and Zhang 2015: fig. 39)	***P.bannaensis***
–	Dorsal connective with weakly developed process subbasally, straight (see Anufriev 1971: fig. 4; Dai and Li 2013: figs 31, 34)	**6**
6	Forewing distinctly marked with brown; aedeagal shaft slender (see Anufriev 1971: fig. 6; Dai and Li 2013: fig. 32)	***P.kurentsovi***
–	Forewing weakly marked with brown; aedeagal shaft not slender (see Dai and Li 2013: fig. 29)	***P.ningxiaensis***
7	Dorsal connective clearly twisted dorsally in lateral aspect (see Hamilton 1980: fig. 62)	***P.tiliae***
–	Dorsal connective clearly twisted ventrally in lateral aspect (Fig. 16)	***P.cudraniae***

### ﻿ Pediopsis albopicta

Taxon classificationAnimaliaHemipteraCicadellidae

Li & Dai sp. nov.

605F33D4-5F1E-5A2B-8146-104FF5BE43E2

https://zoobank.org/75EF8204-6352-4FD0-81AC-965941C9C833

Figs 1–11

#### Examined material.

***Holotype*** ♂, China: Hunan Province, Badagongshan National Natural Reserve, Tianpingshan, 5.viii.2013, collected by Hu Li. ***Paratypes*** 1 ♂, same data as holotype, except 3.viii.2013; 1 ♀, Guizhou Province, Shiqian County, Fodingshan National Natural Reserve, 15.viii.1991, collected by Xiang-Sheng Chen.

#### Description.

**Figures 1–11.**
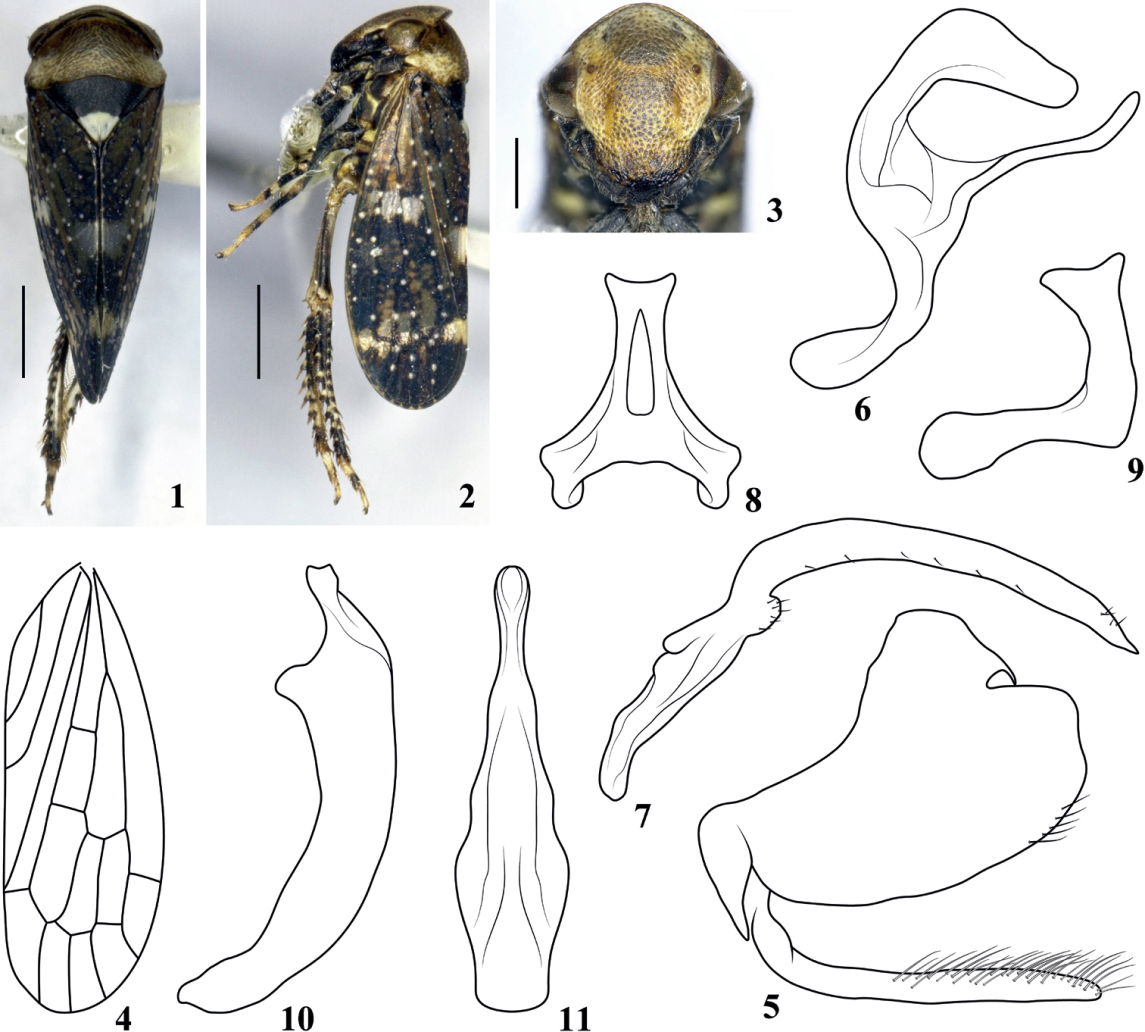
*Pediopsisalbopicta* sp. nov. **1** male habitus, dorsal view **2** male habitus, lateral view **3** face **4** forewing **5** male pygofer and subgenital plate, lateral view **6** dorsal connective, lateral view **7** style, lateral view **8** connective, ventral view **9** connective, lateral view **10** aedeagus, lateral view **11** aedeagus, ventral view. Scale bars: 1 mm (**1, 2**); 0.5 mm (**3**).

***Body color*** (Figs 1–3). Body background color black to dark brown. Head and face (Fig. 3) yellowish, with dark spots or stripes, frontoclypeus slightly milky white, eyes dark brown with reddish tinge, fading to gray; ocelli dark; apex of anteclypeus and gena black. Pronotum (Fig. 1) with anterior half dark brown, posterior half gray, striations on surface darker. Mesonotum (Fig. 1) evenly black with white tip. Forewing (Figs 1, 2) brown, with several transparent patches at midlength and subapically; veins black with clear white spots. Legs yellowish with black or brown patches.

***Body appearance*** (Figs 1–4). Head across eyes (Fig. 1) clearly narrower than pronotum; crown short with anterior and posterior margins almost parallel. Face (Fig. 3) as long as wide across eyes, surface with clear punctures and striations, central region slightly tumid frontally, distance between ocelli relatively large, approximately 8× that from ocellus to adjacent eye. Pronotum (Figs 1, 2) broad, 2.4× wider than long, with strongly oblique striations. Mesonotum (Fig. 1) 1.5× longer than pronotum. Forewing (Figs 1, 2, 4) with three subapical and four apical cells, venation prominent.

***Male genitalia*** (Figs 5–11). Pygofer (Fig. 5) broad basally, lobe short and stout, caudal margin truncated, slightly depressed medially, ventral margin smoothly curved, with scattered marginal setae. Subgenital plate (Fig. 5) slender, of equal width throughout length, with relatively long hair-like setae, its length as long as ventral margin of pygofer. Dorsal connective (Fig. 6) strongly developed, S-shaped, with long slender process produced on ventral margin directed caudad and twisted. Style (Fig. 7) with apophysis stout, angled dorsally at basal 1/3, gradually tapering to pointed apex, with few marginal setae. Connective as in Figs 8, 9. Aedeagus (Figs 10, 11) with basal apodeme and shaft short, the latter shorter than 1/2 length of whole aedeagus, tapered in lateral view to truncate apex, bent dorsally.

#### Measurement.

Body length (including tegmen): 4.3 mm.

#### Distribution.

China (Hunan and Guizhou provinces).

#### Etymology.

The specific epithet of the new species is derived from the Latin words *albus* (white) and *picta* (spot), referring to the white tip of the mesonotum and white spots on the forewing veins.

#### Remarks.

This species can be readily separated from other congeners by the contrasting color pattern of its mesonotum, white spotted forewing veins, and different shape of the aedeagus and dorsal connective.

### ﻿ Pediopsis pianmaensis

Taxon classificationAnimaliaHemipteraCicadellidae

Li & Dai sp. nov.

1FD2945C-2CF7-58AC-B5C9-B6BE42DF7503

https://zoobank.org/F032A413-DF1C-4426-91C5-DE6DF8487C88

Figs 12–21

#### Examined material.

***Holotype*** ♂, China: Yunnan Province, Lushui City, Pianma Town, 26°0'34"N, 98°37'55"E, 26.v.2019, collected by Jia-Jia Wang and Chao Zhang.

#### Description.

**Figures 12–21.**
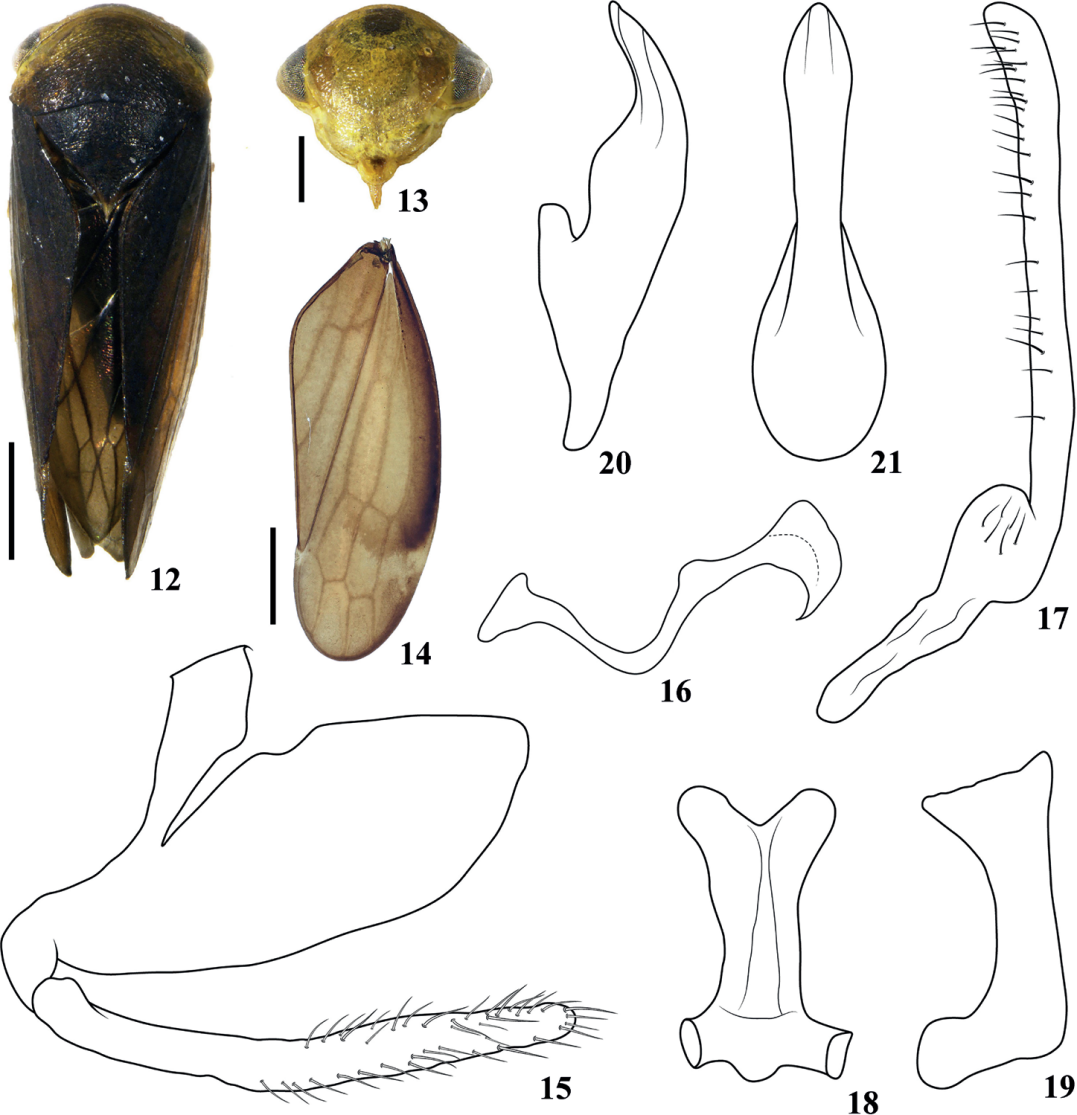
*Pediopsispianmaensis* sp. nov. **12** male habitus, dorsal view **13** face **14** forewing **15** male pygofer and subgenital plate, lateral view **16** dorsal connective, lateral view **17** style, lateral view **18** connective, dorsal view **19** connective, lateral view **20** aedeagus, later view **21** aedeagus, ventral view. Scale bars: 1 mm (**12, 14**); 0.5 mm (**13**).

***Body color*** (Figs 12–14). Specimen from alcohol. Yellowish to dark brown, striations on head, face, and pronotum same color as those of ground color. Head (Figs 12, 13) yellowish, face with dark brown spot at upper central region, eyes brown with gray tinge, ocelli yellow, lower parts of ocelli slightly brown, anteclypeus with brown macula. Pronotum (Fig. 12) yellowish on anterior areas, especially those near eyes, then gradually darkening to almost black at posterior part. Mesonotum (Fig. 12) evenly black, with small yellowish tip. Forewing (Fig. 12) dark brown to almost black on basal part, veins black. Legs yellow with brown markings.

***Body appearance*** (Fig. 12). Head across eyes (Fig. 13) slightly narrower than pronotum; crown short, almost parallel sided. Face (Fig. 13) including eyes 1.2× wider than its length; distance between ocelli nearly 4× that from ocellus to adjacent eye. Pronotum (Fig. 12) 2.2× wider than long, with striations nearly transverse. Mesonotum (Fig. 12) 1.3× longer than pronotum. Forewing (Fig. 14) with two subapical and three apical cells, venation prominent.

***Male genitalia*** (Figs 15–21). Pygofer (Fig. 15) slightly prolonged caudally. Subgenital plate (Fig. 15) slender, slightly longer than ventral margin of pygofer, apical 1/2 with scattered setae. Dorsal connective (Fig. 16) relatively simple, S-shaped with apex broad and tapering to acute ventrally directed tip. Style (Fig. 17) with apophysis relatively straight, slightly widening to truncate apex, with marginal setae. Connective as in Figs 18, 19. Aedeagus (Figs 20, 21) with dorsal apodeme and preatrium short, shaft in lateral view broad basally, thereafter tapered to upturned apex, in ventral view expanded distally with conically rounded apex.

#### Measurement.

Body length (including tegmen): 5.1 mm.

#### Distribution.

China (Yunnan Province).

#### Etymology.

The specific epithet refers to the type locality of the new species, Pianma Town (Yunnan Province), combined with the Latin suffix -*ensis*, meaning “pertaining to”.

#### Remarks.

The new species can be distinguished from all other congeners by its darker body color, forewing with two ante-apical cells, simple aedeagus, and S-shaped dorsal connective.

### ﻿ Pediopsis bannaensis

Taxon classificationAnimaliaHemipteraCicadellidae

Yang & Zhang

079861A7-7264-5D66-BCA3-0F538A96A78A

Pediopsis bannaensis Yang & Zhang, 2015: 488, figs 29–39.

#### Remarks.

This species was described from the holotype and paratype male from China deposited in the Northwest A&F University, Yangling, China (NWAFU) and three male paratypes from Thailand in the Illinois Natural History Survey, Champaign, USA (INHS). However, there are some ambiguities in the original description. Firstly, if the genitalia are drawn accurately, two different species appear to have been figured. The genitalia of one species were shown undissected in fig. 33 of the original description, and based on the aedeagus, the dissected parts of another species were shown in figs 34–39. The aedeagus shown in fig. 33 is the one described, i.e., “Aedeagus strongly tapered from wide base to narrow apex in lateral aspect”. Enquiries made by one of us (Webb) indicate that there are three (not two) Chinese specimens of the species present in the NWAFU collection, with the original type data, all without type labels. Of these specimens only one is dissected and matches fig. 33. Other enquiries made regarding the paratypes in INHS indicate that their aedeagi also match fig. 33. All type series specimens match the habitus images in the original description with respect to general appearance and color pattern, particularly the long dark basal triangles of the mesonotum. However, unaccountably none match the actual specimen imaged based on the leg position in the lateral habitus figure (Yang and Zhang 2015: fig. 30). It is suggested that the dissected specimen in NWAFU be regarded as the holotype even though we do not know what specimen provided the external images, which are of a better specimen.

### ﻿ Pediopsis femorata

Taxon classificationAnimaliaHemipteraCicadellidae

Hamilton

308E94D0-FD92-5F20-8939-006A134C016C

Figs 22–26

Pediopsis femorata Hamilton, 1980: 919.
Pediopsoides femorata –Huang and Viraktamath 1993: 365, misapplication?
Pediopsis femorata –Dai et al. 2018: 188.

#### Remarks.

This species was described based on the female type from Taiwan island, China. Subsequently, Huang and Viraktamath (1993) moved it into *Pediopsoides* Matsumura according to their own specimens from Taiwan. However, Dai et al. (2018) studied the material examined by Hamilton (1980) and Huang and Viraktamath (1993) and considered Huang and Viraktamath’s (1993) identification of *Pediopsoidesfemorata* to be a misidentification and gave it a new name; it may or may not be a new species of *Pediopsoides* (see Li et al. in prep.).

**Figures 22–26.**
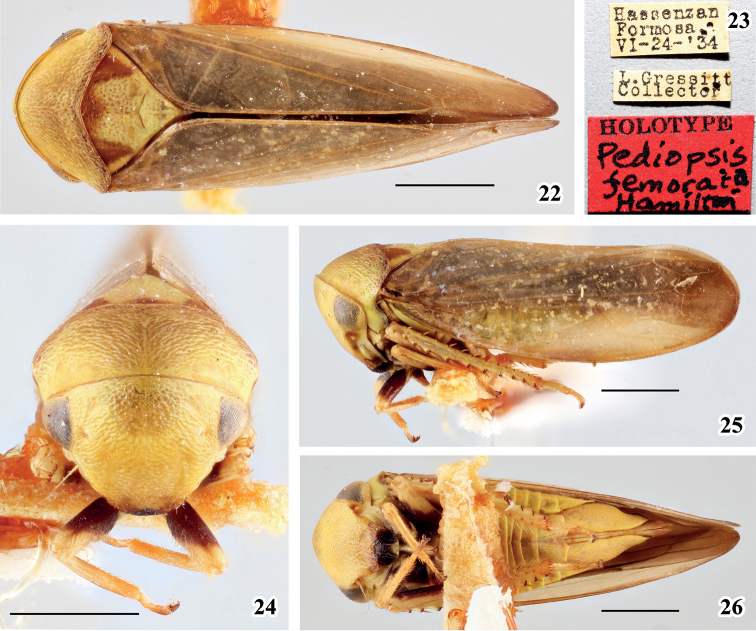
Female holotype of *Pediopsisfemorata* Hamilton **22** habitus, dorsal view **23** labels **24** face, frontal view **25** habitus, lateral view **26** habitus, ventral view. Scale bars: 1 mm. Images © North Carolina State University.

## Supplementary Material

XML Treatment for Pediopsis

XML Treatment for Pediopsis albopicta

XML Treatment for Pediopsis pianmaensis

XML Treatment for Pediopsis bannaensis

XML Treatment for Pediopsis femorata
